# Genomic and Immunological Characterization of Hypermucoviscous Carbapenem-Resistant *Klebsiella pneumoniae* ST25 Isolates from Northwest Argentina

**DOI:** 10.3390/ijms23137361

**Published:** 2022-07-01

**Authors:** Leonardo Albarracin, Ramiro Ortiz Moyano, Juan Martin Vargas, Bruno G. N. Andrade, Juan Cortez Zamar, Stefania Dentice Maidana, Kohtaro Fukuyama, Shoichiro Kurata, María Ángela Jure, Haruki Kitazawa, Julio Villena

**Affiliations:** 1 Laboratory of Immunobiotechnology, Reference Centre for Lactobacilli (CERELA-CONICET), Tucuman 4000, Argentina; lalbarracin@herrera.unt.edu.ar (L.A.); rortiz@cerela.org.ar (R.O.M.); juancorzamar@gmail.com (J.C.Z.); 2 Scientific Computing Laboratory, Computer Science Department, Faculty of Exact Science and Technology, National University of Tucuman, Tucuman 4000, Argentina; 3 Food and Feed Immunology Group, Laboratory of Animal Food Function, Graduate School of Agricultural Science, Tohoku University, Sendai 980-8572, Japan; kotaro.fukuyama.p8@dc.tohoku.ac.jp; 4 Laboratory of Antimicrobials, Institute of Microbiology “Luis C. Verna”, Faculty of Biochemistry, Chemistry and Pharmacy, National University of Tucuman, Tucuman 4000, Argentina; juan.martin.vargas@hotmail.com (J.M.V.); stefi.dentice@gmail.com (S.D.M.); magejure@gmail.com (M.Á.J.); 5 Adapt Centre, Munster Technological University (MTU), T12 P928 Cork, Ireland; bruno.andrade@adaptcentre.ie; 6 Livestock Immunology Unit, International Education and Research Centre for Food and Agricultural Immunology (CFAI), Graduate School of Agricultural Science, Tohoku University, Sendai 980-8572, Japan; 7 Laboratory of Molecular Genetics, Graduate School of Pharmaceutical Sciences, Tohoku University, Sendai 980-8578, Japan; shoichiro.kurata.d5@tohoku.ac.jp

**Keywords:** *Klebsiella pneumoniae*, hypermucoviscous, carbapenem resistant, respiratory infection, genomic, sequence type 25

## Abstract

In recent years, an increase in the prevalence hypermucoviscous carbapenem-resistant *Klebsiella pneumoniae* with sequence type 25 (ST25) was detected in hospitals of Tucuman (Northwest Argentina). In this work, the virulence and the innate immune response to two *K. pneumoniae* ST25 strains (LABACER 01 and LABACER 27) were evaluated in a murine model after a respiratory challenge. In addition, comparative genomics was performed with *K. pneumoniae* LABACER01 and LABACER27 to analyze genes associated with virulence. Both LABACER01 and LABACER27 were detected in the lungs of infected mice two days after the nasal challenge, with LABACER01 counts significantly higher than those of LABACER27. Only LABACER01 was detected in hemocultures. Lactate dehydrogenase (LDH) and albumin levels in bronchoalveolar lavage (BAL) samples were significantly higher in mice challenged with LABACER01 than in LABACER27-infected animals, indicating greater lung tissue damage. Both strains increased the levels of neutrophils, macrophages, TNF-α, IL-1β, IL-6, KC, MCP-1, IFN-γ, and IL-17 in the respiratory tract and blood, with the effect of LABACER01 more marked than that of LABACER27. In contrast, LABACER27 induced higher levels of IL-10 in the respiratory tract than LABACER01. Genomic analysis revealed that *K. pneumoniae* LABACER01 and LABACER27 possess virulence factors found in other strains that have been shown to be hypervirulent, including genes required for enterobactin (*entABCDEF*) and salmochelin (*iroDE*) biosynthesis. In both strains, the genes of toxin–antitoxin systems, as well as regulators of the expression of virulence factors and adhesion genes were also detected. Studies on the genetic potential of multiresistant *K. pneumoniae* strains as well as their cellular and molecular interactions with the host are of fundamental importance to assess the association of certain virulence factors with the intensity of the inflammatory response. In this sense, this work explored the virulence profile based on genomic and in vivo studies of hypermucoviscous carbapenem-resistant *K. pneumoniae* ST25 strains, expanding the knowledge of the biology of the emerging ST25 clone in Argentina.

## 1. Introduction

*Klebsiella pneumoniae* (*K. pneumoniae*) is a Gram-negative pathogen that has developed increasing resistance to carbapenems. These carbapenemase-producing *K. pneumoniae* isolates are resistant to most available antibiotics usually utilized to treat multidrug resistant strains, and therefore, they are now associated with high rates of mortality [1]. In fact, the prevalence of nosocomial infections by carbapenem-resistant *K. pneumoniae* has increased in the hospitalized population, which constitutes a problem of great magnitude due to its high morbidity and mortality. The frequency of carbapenem-resistant *K. pneumoniae* isolates in Argentina, Brazil, Mexico, and Chile has been reported to be 4, 4.9, 7, and 8.9%, respectively; while countries such as Colombia have triple the percentages found in other countries (25.4%) [2]. The Pan American Health Organization (PAHO) reported that despite efforts to control the spread of carbapenem-resistant *K. pneumoniae* strains, the rapid dissemination of these pathogens in Latin America has significantly limited the ability of the national and local public health systems to decrease the associated infections and therefore they still constitute a relevant problem in our region [3].

In addition to multi-resistance to antimicrobials, *K. pneumoniae* is endowed with a set of virulence factors that allow it to produce infectious diseases in various mucosal tissues. Thus, in the last two decades, hypervirulent *K. pneumoniae* strains have emerged as clinically significant pathogens [4,5]. Hypervirulent *K. pneumoniae* strains are typically hypermucoviscous due to the overproduction of their polysaccharide capsule [6]. Hypervirulent and hypermucoviscous *K. pneumoniae* strains are the cause of highly invasive infections, such as liver abscesses, in both healthy and immunosuppressed individuals [5]. Surprisingly, these infections are often complicated by devastating disseminated infections including sepsis and meningitis [4]. Furthermore, unlike infections caused by “classical” *K. pneumoniae*, approximately half of all hypervirulent and hypermucoviscous *K. pneumoniae* infections occur in young, healthy individuals [4,6,7,8].

High-risk clones of carbapenem-resistant and hypermucoviscous *K. pneumoniae* of the sequence types 258 and 11 have spread throughout the world [9], while recent studies reported the appearance of carbapenem-resistant and hypermucoviscous *K. pneumoniae* sequence type 25 (ST25) in Argentina [10,11]. Our own studies conducted in hospitals in the province of Tucuman (northwestern Argentina) showed that the urinary and respiratory tracts were the most common sources of clinical samples with carbapenem-resistant *K. pneumoniae* strains, followed by soft tissues and blood [12,13]. In addition, the high incidence of ST25 clones in our region associated with respiratory and systemic infections emphasizes the importance of implementing epidemiological, genetic, and phenotypic studies of this particular ST [14].

To the best of our knowledge, there are no studies that have characterized the virulence and the inflammatory response induced by *K. pneumoniae* ST25 strains in animal models. Thus, the aim of this work was to develop a murine model of respiratory infection that allows the evaluation of the virulence and immune response against KPC-2-producing hypermucoviscous *K. pneumoniae* ST25. For the study, two multiresistant intrahospital *K. pneumoniae* ST25 strains with different virulence patterns were used. In addition, we aimed to complement these in vivo studies with a comparative genomic analysis to characterize the virulence factors of *K. pneumoniae* ST25 strains and associate them with their infective capacity and the intensity of the inflammatory response induced in the murine model after the respiratory challenge.

## 2. Results

### 2.1. K. pneumoniae LABACER 01 and LABACER 27 Infect the Respiratory Tract of Immunocompetent Adult Mice

**Figure 1:**
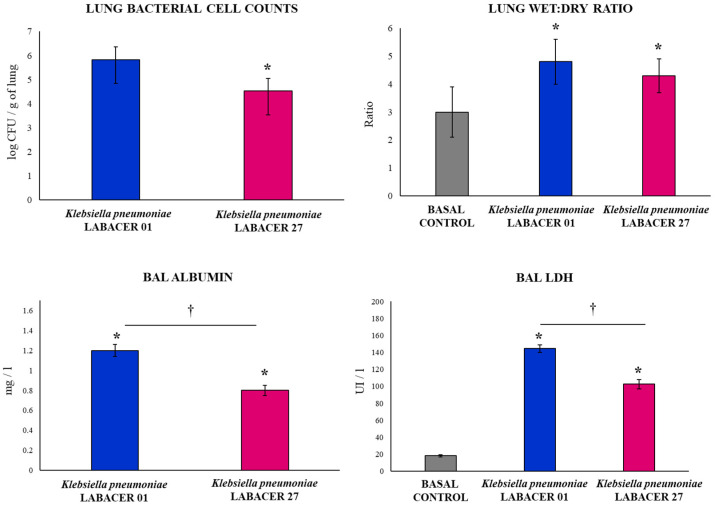
Lung colonization and damage induced by KPC-2-producing hypermucoviscous ST25 strains of *Klebsiella pneumoniae*. Immunocompetent adult BALB/c mice (six weeks) were challenged nasally with *K. pneumoniae* LABACER 01 or LABACER 27. Two days after challenge, the bacterial cell counts in lung homogenates, the lung wet:dry ratio, lactate dehydrogenase (LDH) enzyme activity, and the albumin concentration were determined in broncho-alveolar lavage (BAL) samples. Results represent data from three independent experiments. Asterisks (*) indicate significant differences from the uninfected control group (basal control), *p* < 0.05. The crosses (†) indicate significant differences between the indicated groups, *p* < 0.05. Basal levels of BAL albumin were below the detection limit.

For the experiments, two *K. pneumoniae* isolates were used: LABACER 01 and LABACER 27. These KPC-2-producing hypermucoviscous *K. pneumoniae* ST25 strains were selected based on their virulent capacity [14]. The LABACER 01 strain was recovered from a bone sample of a 20-year-old male patient, and the LABACER 27 strain was isolated from a lung sample of a 63-year-old male patient [12]. The intranasal challenge with both *K. pneumoniae* LABACER 01 and LABACER 27 in BALB/c mice led to significant bacterial burden in the lungs two days after infection (Figure 1). In mice infected with the LABACER 01 strain, the lung bacterial counts were significantly higher compared to the group infected with the LABACER 27 strain (Figure 1). The analysis of bacteria in blood showed that only *K. pneumoniae* LABACER 01 was able to spread from the lungs, as blood cultures in the group of animals infected with LABACER 27 were negative (data not shown).

In order to establish whether the higher bacterial counts in the lungs and the blood spreading induced by *K. pneumoniae* LABACER 01 were correlated with increased tissue damage in the lungs, the lung wet:dry ratio and biochemical studies were performed on broncho-alveolar lavage (BAL) samples from infected mice. Both *K. pneumoniae* LABACER 01 and LABACER 27 significantly increased the lung wet:dry ratio, albumin concentration, and lactate dehydrogenase (LDH) activity in BAL samples compared to non-infected controls (Figure 1). These results indicate that the respiratory challenge of immunocompetent adult mice with KPC-2-producing hypermucoviscous *K. pneumoniae* ST25 strains produced edema and cell damage in the lungs and increased the permeability of the alveolar–capillary barrier. It was also observed that the levels of LDH and albumin in the BAL samples were significantly higher in the group of mice infected with the LABACER 01 strain compared to the animals infected with *K. pneumoniae* LABACER 27, indicating greater virulence of the former strain (Figure 1).

### 2.2. K. pneumoniae LABACER 01 and LABACER 27 Induce a Marked Respiratory and Systemic Inflammatory Response

**Figure 2:**
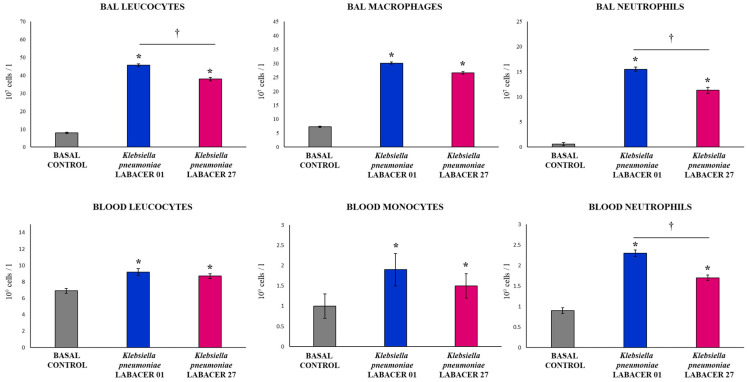
Variations in respiratory and blood leukocytes induced by KPC-2-producing hypermucoviscous ST25 strains of *Klebsiella pneumoniae*. Immunocompetent adult BALB/c mice (six weeks) were nasally challenged with *K. pneumoniae* LABACER 01 or LABACER 27. Two days after challenge, leukocyte, macrophage, and neutrophil counts were performed in broncho-alveolar lavage (BAL) samples, and leukocyte, monocyte, and neutrophil counts were performed in blood. Results represent data from three independent experiments. Asterisks (*) indicate significant differences from the uninfected control group (basal control), *p* < 0.05. The crosses (†) indicate significant differences between the indicated groups, *p* < 0.05.

The total and differential cell counts in the BAL samples showed that the infection with both *K. pneumoniae* LABACER 01 and LABACER 27 increased the values of leukocytes, neutrophils, and macrophages in the respiratory tract when compared to non-infected mice (Figure 2). Significant differences were found between the two groups of infected animals when comparing the total leukocyte counts in the BAL samples. This difference was mainly due to the recruitment of neutrophils since it was observed that animals infected with *K. pneumoniae* LABACER 01 presented levels of these cells in the BAL samples that were significantly higher than those of mice challenged with the LABACER 27 strain (Figure 2). No differences were observed in the number of macrophages in the BAL samples when the LABACER 01 and LABACER 27 groups were compared.

Infection with the *K. pneumoniae* ST25 strains also induced a significant increase in the number of blood leukocytes compared to the uninfected controls (Figure 2). No differences were observed between the LABACER 01 and LABACER 27 groups when the values of total leukocytes and monocytes in blood were analyzed. However, mice challenged with *K. pneumoniae* LABACER 01 had blood neutrophil levels that were significantly higher than those in mice infected with the LABACER 27 strain (Figure 2).

**Figure 3:**
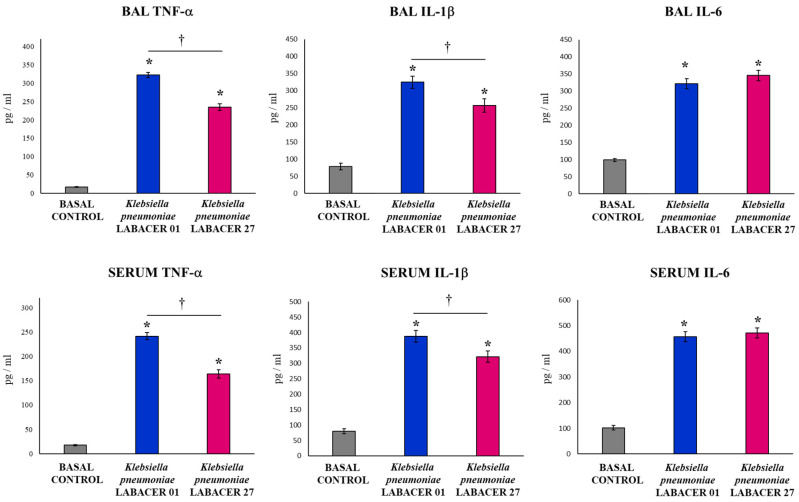
Variations in inflammatory cytokines in the respiratory tract and blood induced by KPC-2-producing hypermucoviscous ST25 strains of *Klebsiella pneumoniae*. Immunocompetent adult BALB/c mice (six weeks) were nasally challenged with *K. pneumoniae* LABACER 01 or LABACER 27. Two days after challenge, the TNF-α, IL-1β, and IL-6 levels were determined in broncho-alveolar lavage (BAL) and blood samples. Results represent data from three independent experiments. Asterisks (*) indicate significant differences from the uninfected control group (basal control), *p* < 0.05. The crosses (†) indicate significant differences between the indicated groups, *p* < 0.05.

The intranasal challenge with the *K. pneumoniae* ST25 strains induced a marked increase in the levels of the inflammatory cytokines TNF-α, IL-1β, and IL-6 in both BAL and serum samples compared to uninfected controls (Figure 3). No differences were observed between the LABACER 01 and LABACER 27 groups when the IL-6 levels were studied in the serum and BAL samples. However, mice infected with *K. pneumoniae* LABACER 01 presented significantly higher levels of TNF-α and IL-1β than animals challenged with the LABACER 27 strain, in both serum and BAL samples (Figure 3).

**Figure 4:**
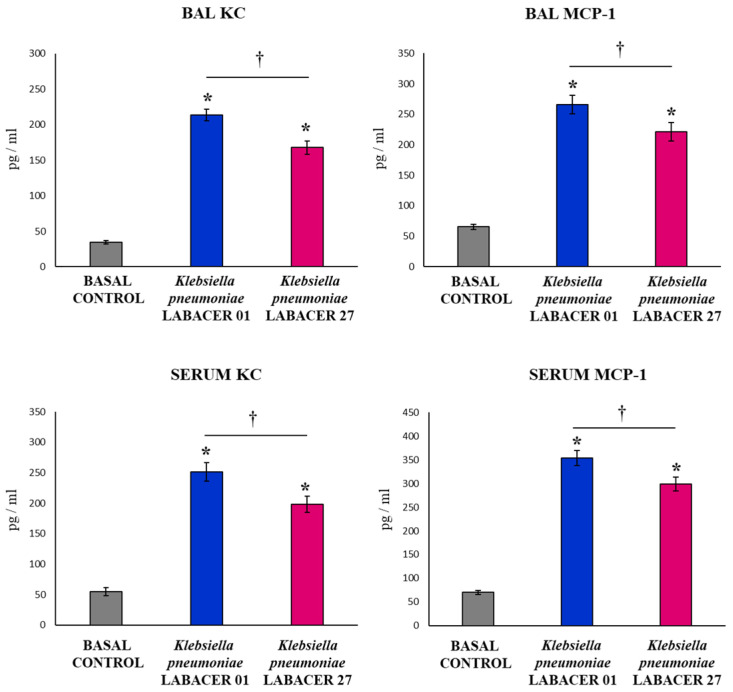
Variations in the inflammatory chemokines in the respiratory tract and blood induced by KPC-2-producing hypermucoviscous ST25 strains of *Klebsiella pneumoniae*. Immunocompetent adult BALB/c mice (six weeks) were nasally challenged with *K. pneumoniae* LABACER 01 or LABACER 27. Two days after challenge, the KC and MCP-1 levels were determined in broncho-alveolar lavage (BAL) and blood samples. Results represent data from three independent experiments. Asterisks (*) indicate significant differences from the uninfected control group (basal control), *p* < 0.05. The crosses (†) indicate significant differences between the indicated groups, *p* < 0.05.

The respiratory infections induced by *K. pneumoniae* LABACER 01 or LABACER 27 increased the levels of the inflammatory chemokines KC (murine homologue of IL-8) and MCP-1 in both the respiratory tract and blood (Figure 4). The two chemokines had significantly higher concentrations in mice challenged with the LABACER 01 strain compared to animals infected with *K. pneumoniae* LABACER 27, in both serum and BAL samples.

**Figure 5:**
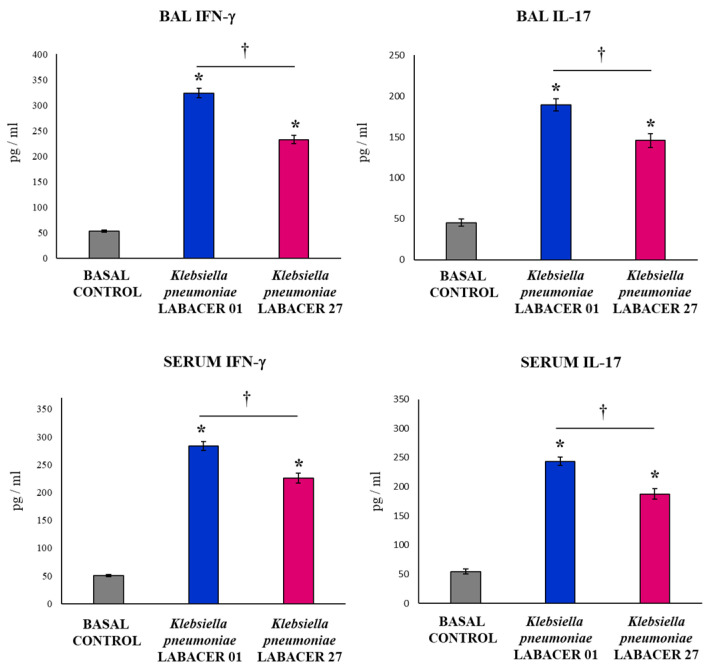
Variations in the inflammatory cytokines in the respiratory tract and blood induced by KPC-2-producing hypermucoviscous ST25 strains of *Klebsiella pneumoniae*. Immunocompetent adult BALB/c mice (six weeks) were nasally challenged with *K. pneumoniae* LABACER 01 or LABACER 27. Two days after challenge, the IFN-γ and IL-17 levels were determined in broncho-alveolar lavage (BAL) and blood samples. Results represent data from three independent experiments. Asterisks (*) indicate significant differences from the uninfected control group (basal control), *p* < 0.05. The crosses (†) indicate significant differences between the indicated groups, *p* < 0.05.

The variations in the levels of IFN-γ and IL-17 in response to the nasal challenge with the *K. pneumoniae* ST25 strains were also investigated. The infection increased the concentrations of both immunological factors in the BAL and serum samples relative to the control animals (Figure 5). Mice infected with *K. pneumoniae* LABACER 01 presented respiratory and serum levels of IFN-γ and IL-17 that were significantly higher than those of animals challenged with the LABACER 27 strain (Figure 5).

**Figure 6:**
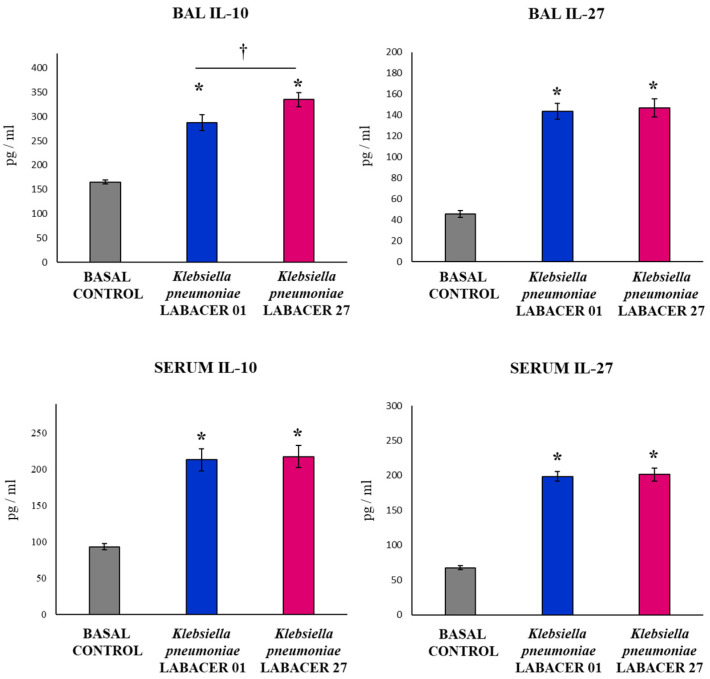
Variations in the regulatory cytokines in the respiratory tract and blood induced by KPC-2-producing hypermucoviscous ST25 strains of *Klebsiella pneumoniae*. Immunocompetent adult BALB/c mice (six weeks) were nasally challenged with *K. pneumoniae* LABACER 01 or LABACER 27. Two days after challenge, the IL-10 and IL-27 levels were determined in broncho-alveolar lavage (BAL) and blood samples. Results represent data from three independent experiments. Asterisks (*) indicate significant differences from the uninfected control group (basal control), *p* < 0.05. The crosses (†) indicate significant differences between the indicated groups, *p* < 0.05.

The levels of two regulatory cytokines, IL-10 and IL-27, were also determined in serum and BAL samples from mice infected with the *K. pneumoniae* ST25 strains (Figure 6). Both pathogens induced significant increases in IL-10 and IL-27 levels in BAL and serum samples compared to uninfected animals. No differences were observed between the *K. pneumoniae* LABACER 01 and LABACER 27 groups when serum IL-10 and the levels of IL-27 in the BAL and serum samples were studied. However, mice infected with *K. pneumoniae* LABACER 27 had significantly higher IL-10 levels in the BAL samples than animals challenged with the LABACER 01 strain (Figure 6).

### 2.3. Genomic Characterization of K. pneumoniae LABACER 01 and LABACER 07

We previously reported that the analysis of the *K. pneumoniae* LABACER 01 genome revealed a size of 5,598,020 bp with 5608 coding sequences and the presence of 19 antimicrobial resistance genes [14]. On the other hand, the LABACER 27 genome analysis showed a size of 5,622,382 bp with 5580 coding sequences and the presence of 20 different antimicrobial resistance genes [14]. In addition, we showed that other general genomic characteristics of the LABACER 01 and 27 strains such as GC% and numbers of tRNAs and rRNAs agreed with those published for other *K. pneumoniae* strains. Here, we used the complete genome sequences of *K. pneumoniae* LABACER 01 and LABACER 27 to characterize their virulence factors.

**Figure 7:**
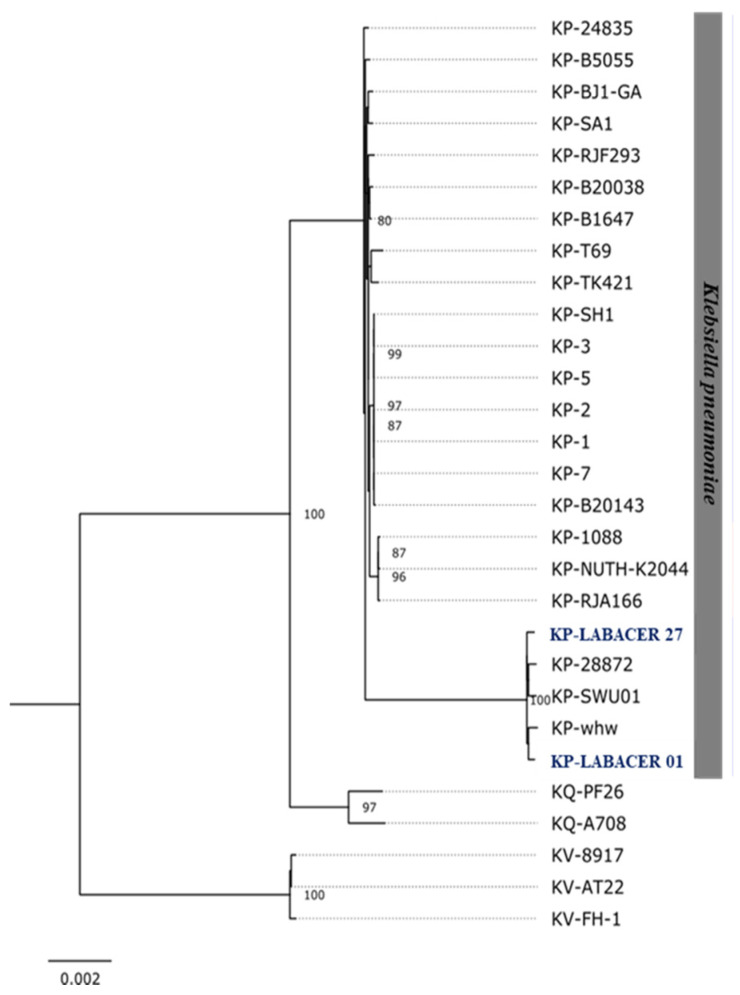
Phylogenetic tree constructed with sequences of genes encoding ribosomal protein subunits (rps) of *Klebsiella pneumoniae* (KP), *Klebsiella quasipneumoniae* (KQ), and *Klebsiella variicola* (KV).

The 32 ribosomal protein subunit (rps)-coding genes identified in the sister species, *K. pneumoniae*, *K. quasipneumoniae*, and *K. variicola*, were used to generate a phylogenomic tree with a robust and well-defined topology in which isolates of *K. variicola* species were found to be automatically defined as outgroups (Figure 7). Within the *K. pneumoniae* group, the hypermucoviscous strains LABACER 01 and LABACER 27, as well as strains 28876, SWU01, and KP-whw, formed a separate phylogenomic group from other *K. pneumoniae* strains (Figure 7).

**Figure 8:**
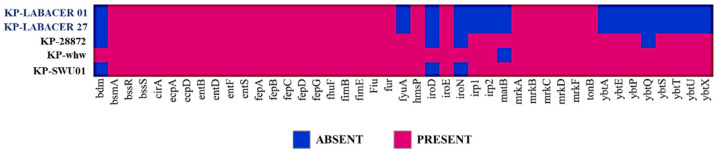
Virulence factor genes shared between the *Klebsiella pneumoniae* LABACER 01 (KP-LABACER 01), LABACER 27 (KP-LABACER 27), 28872, whw, and SWU01 strains.

Considering the results of the phylogenetic analysis based on the rps sequences, we decided to compare the virulence-related genes of the LABACER 01 and LABACER 27 strains with *K. pneumoniae* SWU01, Kp-whw (or whw) and 28872 (or KP28872) (Figure 8). *K. pneumoniae* SWU01 is an ST11 strain isolated from the blood of an intensive care unit patient with severe pulmonary infection [15], which has the virulence genes *iroBCDN*, *rmpA*, *iutABC*, *kfu*, *allS*, *pagO*, *irp*, *ybtS*, *shiF*, *fimH,* and *mrkD* in its genome [16]. *K. pneumoniae* whw is a ST1049 strain isolated from a neck abscess [17], which was characterized as having the *rmpA*, *rmpA2*, *iuc* (aerobactin), *iro* (salmokelin), *hmsP*, *bssR*, *bdm*, *bsmA,* and *bssS* genes. *K. pneumoniae* 28872 is an ST125 strain isolated from the urinary tract [18], with the genes *ecp*, *entABCDEF*, *fepABCDG*, *fes*, *ybdA*, *iroE*, *ybtAEPSTUX*, *irp1*, *irp2*, and *fyuA,* and for the fimbriae, *fimABCDEFGHIK* and *mrkABCDFHIJ,* in its genome [19].

The virulence factor analysis of the genomes of *K. pneumoniae* LABACER 01 and LABACER 27 in relation to the WU01, whw, and 28872 strains revealed that the five bacteria share most of the virulence genes. The most notable exceptions were the absence of the *fyuA*, *irp1*, *irp2,* and *ybtAEPQSTUX* genes in the LABACER strains that are present in the other genomes (Figure 8).

**Figure 9:**
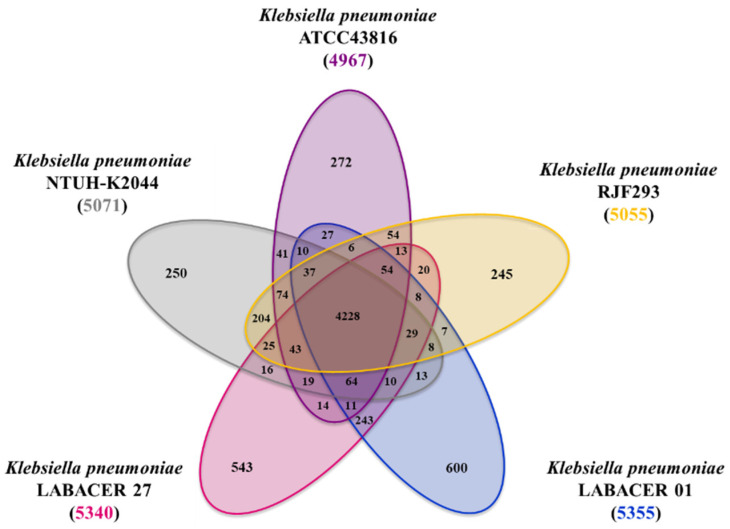
Venn diagram comparing shared genes and unique genes found in the sequenced genomes of *Klebsiella pneumoniae* strains LABACER 01, LABACER 27, NTUH-K2044, ATCC43816, and RJF293.

In addition, we performed a comparative genomic analysis of *K. pneumoniae* LABACER 01 and LABACER 27 with the isolates that were shown to efficiently colonize and infect mice under experimental conditions: NTUH-K2044 [20,21], ATCC43816 [22,23,24,25,26], and RJF293 [27]. Genome analysis of the hypervirulent ST493 *K. pneumoniae* strain ATCC 43816 showed that genes such as *ompK36*, *tolABQ*, *lpp*, *bamB*, *yfgL*, *surA*, *rffG*, and *wecA* are important for its infectivity [25]. The hypermucoviscous and hypervirulent strain NTUH-K2044 has been shown to possess the virulence factors *magA*, *rmpA*, *vagCD*, and *iroNBCD,* as well as the *ybt* genes [26], while the hypervirulent strain *K. pneumoniae* RJF293, which is ST374, possesses 69 genes of virulence factors including the *fep, ent, yag, mrk, lpx,* and *kfu* genes [27]. As shown in the Venn diagram (Figure 9), the five *K. pneumoniae* strains shared a core of 4228 genes. Among the virulence genes shared by the five *K. pneumoniae* strains were the major subunit of the common pilus EcpA (*ecpA*), the fimbrial chaperone EcpB (*ecpB*), the outer membrane protein EcpC (*ecpC*), the fimbrial adhesin EcpD (*ecpD*), and the fimbrial chaperone EcpE (*ecpE*). Type 1 fimbrial biosynthesis-associated genes including fimbrial protein A chain type 1 (*fimA1*) and outer membrane protein FimD (*fimD1* and *fimD4*) were also detected. The genomes of the five strains presented the genes for the fimbrial subunit LpfA (*lpfA2*), the fimbrial chaperone LpfB (*lpfB1*), fimbrial adhesive proteins (*mrkD1* and *mrkD2*), the fimbrial protein YcbV (*ycbV1*), as well as the fimbrial chaperones YadV (*yadV3*) and YraI (*yraI1*).

*K. pneumoniae* LABACER 01 and LABACER 27 also shared genes of toxin–antitoxin systems including TabA (*tabA*), YefM (*yefM*), HigA-2 (*higA2*), HipB (*hipB*), and HipA (*hipA*) with strains NTUH-K2044, ATCC43816, and RFJ293. Among the core virulence genes, the sensor protein PhoQ (*phoQ*) and the transcriptional regulator proteins of virulence PhoP (*phoP1* and *phoP2*) were also detected, which are members of the two-component regulatory system PhoP/PhoQ that regulates gene expression involved in virulence, adaptation to acidic environments, and resistance to host antimicrobial peptides. The virulence regulator VirF (*virF*) was also found to be a shared gene among the five strains. This regulator activates the transcription of virG and virB, which are activators of the virulence regulator ipaABCD.

Furthermore, it was observed that the LABACER 01 and LABACER 27 strains presented 600 and 543 unique genes, respectively, when simultaneously compared with *K. pneumoniae* NTUH-K2044, ATCC43816, and RFJ293 (Figure 9). Among the unique genes of *K. pneumoniae* LABACER 01 (Table S1) was the outer membrane protein C (*ompC*), which forms pores that allow passive diffusion of small molecules through the outer membrane. In *K. pneumoniae*, it has been shown to bind to the C1q component and activate the classical pathway of the complement system [2]. The FimC chaperone protein (*fimC1*), S-fimbrial protein subunit SfaG (*sfaG1*), fimbrial protein A (*smfA*), fimbrial minor pilin PAP (*papH*), outer membrane accommodating protein PapC (*papC*), fimbrial chaperone YfcS (*yfcS*), fimbrial protein YfcR (*yfcR*), and cytoskeleton-binding toxins CbtA (*cbtA*) and CbeA (*cbeA*) were also found as unique genes in the LABACER 01 strain genome.

Among the unique genes of *K. pneumoniae* LABACER 27 (Table S2), we found the VapB antitoxin (*vapB*), the HigA antitoxin (*higA*), the pilin (*traA*), the biofilm growth-associated repressor (*bigR*), the structural major subunit pilus (*bfpA*), the fimbrial chaperone YadV (*yadV2* and *yadV3*), the fimbrial protein SfaA (*sfaA*), the fimbrial chaperone SfmC (*sfmC*), and the accommodating outer membrane protein SfmD (*sfmd*) that is part of the fimbrial operon *sfmACDHF*. The *rfaH*, *copA*, *aroE*, *ilvD* and *purH* genes were also detected among the unique genes in the LABACER 27 strain genome.

The comparative analysis also revealed that the LABACER 01 and LABACER 27 strains share only 243 genes between them as observed in the Venn diagram (Figure 9). Among these genes, virulence factors associated with the MazE type II toxin–antitoxin system were found.

### 2.4. Morphological Characterization of K. pneumoniae LABACER 01 and LABACER 27

**Figure 10:**
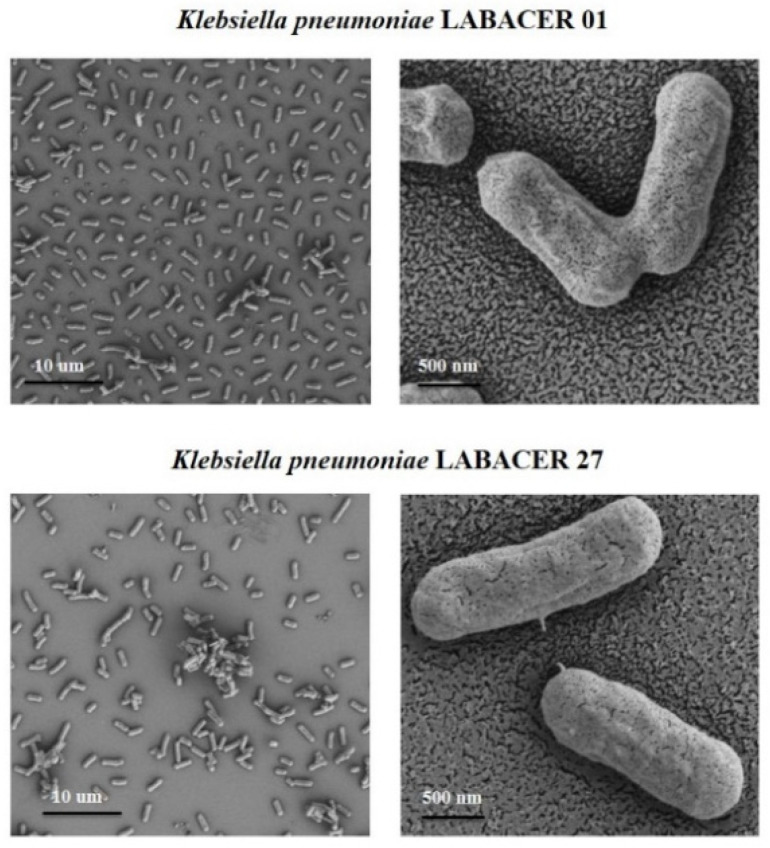
Scanning electron microscopy analysis of the KPC-2-producing hypermucoviscous ST25 *Klebsiella pneumoniae* strains LABACER 01 and LABACER 27.

**Figure 11:**
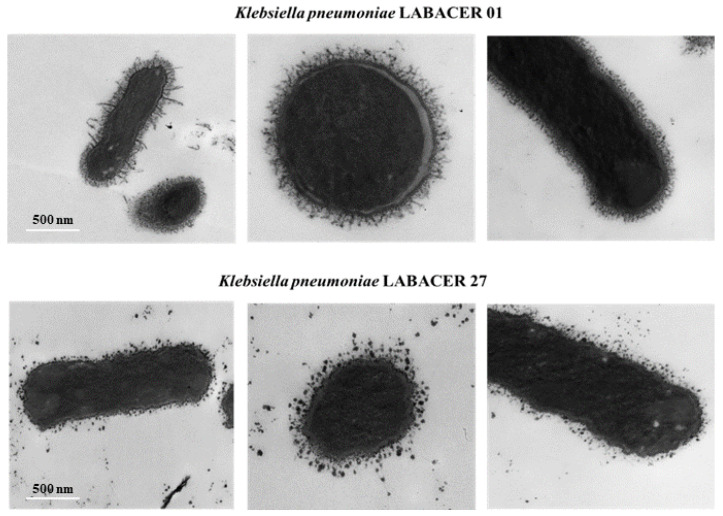
Transmission electron microscopy analysis of the KPC-2-producing hypermucoviscous ST25 *Klebsiella pneumoniae* strains LABACER 01 and LABACER 27.

Finally, we performed electron microscopy analysis to characterize the morphology of *K. pneumoniae* LABACER 01 and LABACER 27. The morphology of *K. pneumoniae* LABACER 01 and LABACER 27 was studied using scanning electron microscopy (Figure 10) and transmission electron microscopy (Figure 11). Both strains appeared as bacilli with sizes between 1 and 2 μm long and 0.6 to 0.8 μm wide. Multicellular aggregate formation was observed more frequently in cultures of *K. pneumoniae* LABACER 27 compared to cultures of strain LABACER 01 (Figure 10). In addition, clear differences were observed in cell structures that projected from the bacterial surface (Figure 11).

## 3. Discussion

An increase in KPC-2-producing hypermucoviscous *K. pneumoniae* strains belonging to ST25 has been detected in northwestern Argentina in recent years [12,13]. The high incidence of the ST25 clone in our region associated with respiratory and systemic infections emphasizes the importance of implementing more genetic and phenotypic studies of this particular ST [14]. Thus, the first objective of this work was to evaluate the virulence of two strains of *K. pneumoniae* ST25 isolated from hospitalized patients in a murine model. To assess their virulence, 6-week-old BALB/c mice were nasally challenged with 10^7^ CFUs of LABACER 01 or LABACER 27, and colonization and lung damage, as well as respiratory immunity, were assessed after infection. Both *K. pneumoniae* LABACER 01 and LABACER 27 were detected in the lungs of infected mice, with higher counts for LABACER 01. In addition, it was observed that only LABACER 01 was detected in the blood cultures in the post-infection time studied. The evaluation of lung damage also revealed that both *K. pneumoniae* strains induced cellular damage and altered of the alveolar–capillary barrier, with this effect being more marked for LABACER01. These results allowed us to conclude that both hypermucoviscous KPC-2-producing *K. pneumoniae* ST25 strains are virulent and capable of infecting the respiratory tract of immunocompetent adult mice and that the LABACER 01 strain is more virulent than *K. pneumoniae* LABACER 27.

Our findings are in line with other studies carried out in mice in which the ability of certain strains of *K. pneumoniae* to infect experimental animals when administered intranasally has been demonstrated. In this regard, one of the most widely used strains is *K. pneumoniae* ATCC 43816 as it recapitulates acute pneumonia with fatal systemic spread at a relatively low infectious dose (10^4^–10^5^ CFUs intranasally) when administered to adult C57BL/6 [22,23,24,28] or BALB/c [29,30] mice. On the other hand, studies carried out in immunocompetent mice infected with the clinical isolates *K. pneumoniae* KPC+ or OXA-48+ showed that doses of 10^7^ CFUs or higher are necessary to achieve a respiratory infection that reaches densities of 6–7 log_10_ CFUs/g lung [31]. Similarly, doses of 10^8^ CFUs were required to achieve respiratory infections in adult C57BL/6 [32] and BALB/c [33] mice with clinical isolates of *K. pneumoniae*. Consistent with these studies, doses of 10^7^ CFUs or higher of LABACER 01 or LABACER 27 were required in this study to achieve respiratory infection in adult immunocompetent BALB/c mice. Therefore, the first contribution of this work is the successful development of an experimental model in mice that allows the examination of the respiratory infection produced by KPC-2-producing hypermucoviscous *K. pneumoniae* strains belonging to ST25.

The evaluation of the respiratory innate immune response showed that both *K. pneumoniae* LABACER 01 and LABACER 27 increased the levels of neutrophils, monocytes/macrophages, TNF-α, IL-1β, IL-6, KC, MCP-1, IFN-γ, and IL-17 in the respiratory tract and blood. Our results agree with the findings reported in adult C57BL/6 mice nasally challenged with *K. pneumoniae* ATCC 43816 in which a significant increase in the levels of TNF-α and IL-6 was observed in the respiratory tract of infected animals [22]. Subsequent studies demonstrated a pulmonary infiltration with CCR2^+^ cells (monocytes and neutrophils) that was maintained for 48 h in response to nasal challenge with the pathogen [23]. Furthermore, the work showed that CCR2^+^ monocytes were the main producers of TNF-α in infected mice. Work carried out in mice challenged with clinical isolates of *K. pneumoniae* also showed increases in the pulmonary levels of TNF-α, IL-1β, IL-6 [32], CXCL1, MCP-1, and MIP-2 [33], as well as neutrophil infiltration in the respiratory tract [32,33].

The respiratory and systemic inflammatory response was significantly higher in animals infected with *K. pneumoniae* LABACER 01 compared to those challenged with LABACER 27. Studies in experimental animals show that the inflammatory response can have both a positive and negative role in the resistance to respiratory infection by *K. pneumoniae*. It was reported that antibiotic-treated C57BL/6 adult mice are more susceptible to infection with *K. pneumoniae* ATCC 43816 compared to control animals without antibiotic therapy [22]. Interestingly, antibiotic-treated animals exhibited alveolar macrophages with significantly decreased bactericidal activities compared to controls, as well as decreased expressions of IL-6 and TNF-α in the lung. In addition, it was recently described that the administration of IL-33 is capable of increasing the protection of BALB/c mice against respiratory infection by *K. pneumoniae* [30]. This effect was associated with enhanced recruitment of inflammatory neutrophils and monocytes to the airways. It was also reported that adult C57Bl/6J mice with the CD36^-/-^ phenotype (scavenger receptor expressed predominantly in macrophages) have a higher pulmonary bacterial load and greater extrapulmonary dissemination after respiratory infection induced with hypermucoviscous *K. pneumoniae* [34]. The greater susceptibility of CD36^-/-^ mice to *K. pneumoniae* infection was associated with a lower phagocytic activity of macrophages as well as a decrease in the production of the inflammatory cytokines IFN-γ, IL-1β, IL-6, IL-12p70, IL-17A, MCP-1, and TNF-α. These results clearly indicate that the inflammatory response is necessary to control the colonization of *K. pneumoniae* in the respiratory tract. In contrast, it was reported that a combined antibiotic treatment can protect mice from severe pneumonia induced by *K. pneumoniae* ATCC 43816 and that this beneficial effect is associated with the reduction of the accumulation of inflammatory cells in the lungs [24]. Antibiotic treatment decreased NF-κB and caspase-1/NLRP3 inflammasome signaling pathways, attenuating abnormally elevated levels of TNF-α, IL-1β, IL-6, and IL-17, thereby increasing the survival of infected mice. Together, these findings indicate that protection against respiratory infection caused by *K. pneumoniae* is achieved by inducing an inflammatory response capable of eliminating the pathogen, which must be efficiently regulated to prevent inflammatory damage and preserve lung function, as has been described for other bacterial [35,36] and viral [37,38] respiratory pathogens. Kinetics studies of inflammatory cells and factors both in the respiratory tract and blood would be of value to understand why, even though LABACER01 induced a more notorious inflammatory response than LABACER27, it was the strain with the greatest capacity to colonize the lung and spread into the blood.

**Figure 12:**
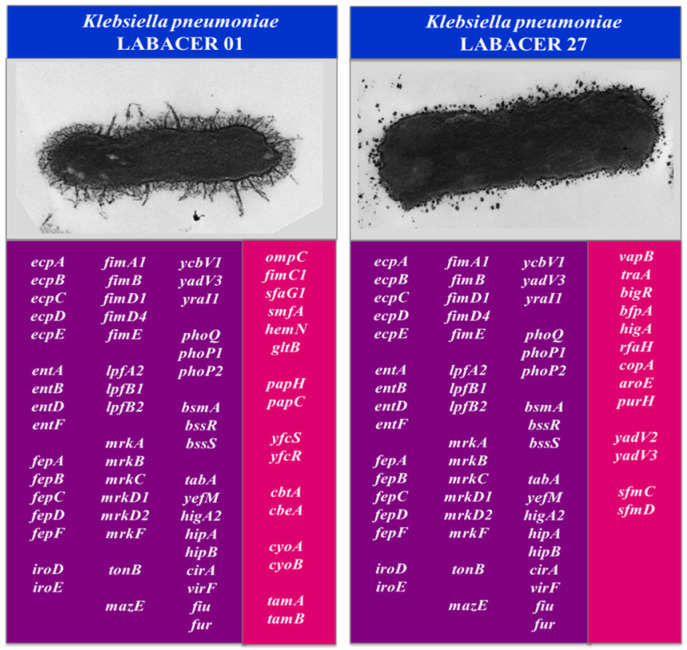
Virulence factor genes found in KPC-2-producing hypermucoviscous ST25 *Klebsiella pneumoniae* strains LABACER 01 and LABACER 27. The genes in the purple boxes are shared between the two LABACER strains as well as with other hypermucoviscous and hypervirulent *K. pneumoniae* strains (28876, SWU01, whw, ATCC 43816, NTUH-K2044, and RJF293). The genes in the pink boxes were detected only in the indicated LABACER strain.

The second objective of this work was to perform a genome analysis of the LABACER 01 and LABACER 27 strains to identify genes associated with virulence and colonization. To characterize these virulence factors, a comparative analysis was first carried out using the genomes of the hypermucoviscous strains LABACER 01 and LABACER 27, as well as the 28876, SWU01, and whw strains, which formed a phylogenomic group separate from other *K. pneumoniae* strains by using the 32 rps-encoding genes. *K. pneumoniae* 28876, SWU01, and whw are hypermucoviscous and hypervirulent bacteria, although with different STs [15,16,17,19]. The genomes of *K. pneumoniae* LABACER 01 and LABACER 27 were also compared with those of the strains ATCC 43816 [21,22,23], NTUH -K2044 [21,22], and RJF293 [27], which were shown to efficiently colonize and infect mice under experimental conditions. This comparative genomic analysis made it possible to define the groups of virulence factor genes present in the hypermucoviscous KPC-2-producing *K. pneumoniae* ST25 strains used in this work, as shown in Figure 12.

Our results indicate that *K. pneumoniae* LABACER 01 and LABACER 27 possess virulence factors found in other strains that have been shown to be hypervirulent, including genes necessary for the biosynthesis (*entABCDEF*) and transport (*fepABCDG*) of enterobactin [39] as well as for the biosynthesis of salmochelin (*iroDE*) [40]. In both strains, the toxin–antitoxin system genes *tabA*, *yefM*, *higA2*, *hipB*, and *hipA* were also detected, as well as the genes that regulate the expression of virulence factors, i.e., *phoQ*, *phoP1*, *phoP2,* and *virF*. LABACER strains also possess the adhesion factor genes *ecpABCDE*, *fimA1BD1D4E*, *lpfA2*, *lpfB1*, *mrkABCD1D2E*, *ycbV1*, *yadV3,* and *yraI1*. This set of virulence factors would allow both strains to efficiently infect the respiratory tract of immunocompetent adult mice, as demonstrated by our in vivo studies. For example, salmochelin has been shown to increase the ability of *K. pneumoniae* to colonize the nasopharynx [41]. Furthermore, studies have reported that salmochelin allows nasopharyngeal colonization of *K. pneumoniae* in immunocompetent hosts. It was described that this virulence factor is only present in 2–4% of strains of nosocomial *K. pneumoniae*, but it is much more frequent in hypervirulent strains [39,42,43]. On the other hand, the presence of siderophores such as enterobactin and salmochelin not only help *K. pneumoniae* to sequester iron but also stimulate the inflammatory response and favor the spread of the pathogen from mucosal surfaces. Siderophores sequester iron from respiratory epithelial cells but also induce the secretion of cytokines such as IL-8, IL-6, and CCL20 in vivo [44] and activate transcription factors that control vascular permeability and the expression of inflammatory genes [44], promoting the dissemination of bacteria from the lungs to the spleen [45]. Studies in which the purified fimbriae mrk were used to immunize experimental animals showed that the specific immune response against these proteins significantly increases the resistance of mice to respiratory challenge with virulent *K. pneumoniae* strains [46], emphasizing the significant role of the fimbriae in the respiratory infection caused by this pathogen.

In the LABACER strains, *ybt* or *iuc* genes were not detected, which code for yersinibactin and aerobactin, respectively [40]. The siderophore ybt has been associated with respiratory tract infections in patients and is sufficient to promote pneumonia in a mouse model [43,47]. In addition, comparative studies carried out in mice and using aerobactin-producing and non-producing *K. pneumoniae* strains showed that animals infected with the iuc^+^ strain had lower survival and greater lung tissue damage compared to mice challenged with *K. pneumoniae* iuc^−^ [48]. The lack of these virulence factors would explain why higher doses of LABACER strains (10^7^ CFUs) are necessary to induce infections in the respiratory tract of experimental animals compared to other *K. pneumoniae* such as ATCC 43816 [19,48] and NTUH-K2044 [26] that do have the *ybt* and *iuc* genes in their genomes.

Comparative genomics studies performed in this work also showed that the LABACER 01 and LABACER 27 strains possess unique virulence factors when compared to each other (Figure 12). *K. pneumoniae* can remodel its outer membrane in response to stress to maintain its integrity as a barrier and thus promote its survival in the infected host. This property has been associated with the presence of the *tamA* and *tamB* genes [49,50]. Furthermore, it has been shown that *K. pneumoniae* strains deficient in the *tamA* gene are cleared more rapidly in respiratory-challenged mice. Animals challenged with *tamA*-deficient mutants had longer survival, lower bacterial counts in the lung, and less blood shedding compared to mice challenged with *tamA*-carrying *K. pneumoniae* [49]. Therefore, the presence of *tamA* in the genome of LABACER 01 and not in LABACER 27 could be associated with the ability of the former to colonize the lungs and spread to the blood of infected mice more efficiently. *K. pneumoniae* LABACER 01 also possesses the genes for fimbriae, i.e., *fimC1*, *sfaG1, smfA, papH, papC, yfcS,* and *yfcR*. SMF fimbriae were first described in *Serratia marcescens* strains, and the genomic region encoding the fimbriae was later identified to be composed of *smf* genes [51] and to be present in some strains of *Klebsiella* [52]. This fimbria has been associated with adhesion and persistence in urinary epithelium [53,54]. These findings allowed us to predict that *K. pneumoniae* LABACER 01 could behave as an important pathogen of the urinary tract. More functional and genetic studies are needed to explore this possibility.

*K. pneumoniae* LABACER 27 possesses the fimbriae genes *yadV2*, *yadV3,* and *bfpA* (Figure 12). The Yad fimbria has been associated with the ability of pathogenic *E. coli* strains to colonize abiotic surfaces, as well as to adhere to epithelial cells and even inhibit the phagocytic activity of macrophages [55]. The *bfp* operon encodes a BfpA pilin that belongs to type IVB fimbriae and that has been widely characterized in enteropathogenic *E. coli* strains for its ability to promote adhesion to epithelial cells [56]. Recent studies reported that BfpA is also capable of activating the innate immune response mediated by macrophages by stimulating their production of TNF-α, IL-1β, IL-6, IL-12, and MCP-1 [57]. Interestingly, the work also showed that higher concentrations of BfpA can induce IL-10 production by macrophages, and it was suggested that this could be a mechanism to regulate the inflammatory response in favor of the pathogen. Our genomic study also detected the presence of the *rfaH*, *copA,* and *aroE* genes in the *K. pneumoniae* LABACER 27 genome. In an interesting functional genomic study by Bachman et al. [58], genes that are of importance to *K. pneumoniae* for efficient lung infection were identified. The researchers identified more than 300 genes that are required by *K. pneumoniae* to induce pneumonia in a mouse model. Among these genes were *rfaH* and *aroE*, which are necessary to resist the microbicidal action of the complement system. It was also detected that the *copA* gene is necessary to prevent the bactericidal effect of copper [58].

The difference in the virulence factors present in the genomes of the LABACER 01 and LABACER 27 strains could also explain the different magnitude in the activation of the respiratory and systemic inflammatory response observed in our in vivo studies. For example, *K. pneumoniae* LABACER 01 possesses the *ompC* gene that binds to the C1q component and activates the classical pathway of the complement system [2], while the LABACER 27 strain possesses the *rfaH* genes and *aroE* that reduce complement action [58]. The LABACER 27 strain also has the genes *bfpA* and *yad* that would help modulate the inflammatory response in the respiratory tract, favoring its persistence.

## 4. Materials and Methods

### 4.1. K. pneumoniae LABACER 01 and LABACER 27

*K. pneumoniae* LABACER 01 and LABACER 27 were isolated at the “Angel Cruz Padilla” hospital in the city of San Miguel de Tucuman (Tucuman, Argentina). Both hypermucoviscous carbapenem-resistant *K. pneumoniae* ST25 strains were isolated from the intensive care unit and were selected based on their virulent capacity [14]. The LABACER 01 strain was recovered from a bone sample of a 20-year-old male patient, and the LABACER 27 strain was isolated from the lung sample of a 63-year-old male patient [12]. Both microorganisms were identified by matrix-assisted laser desorption/ionization (MALDI-TOF) (Microflex LT; Bruker Daltonik GmbH, Bremen, Germany) and kept in the Culture Collection of the Certified Bacteriology Laboratory (LABACER, National University of Tucuman, Tucuman, Argentina) [14].

### 4.2. Murine Infection Model

All experiments were performed in accordance with the guide for the care and use of laboratory animals and were approved by the CERELA-CONICET Animal Care and Ethics Committee under the BIOT-CRL/19 protocol. Six-week-old male BALB/c mice were used, obtained from the closed colony maintained in the CERELA Animal Facility (Reference Center for Lactobacilli, CONICET, Tucuman, Argentina). The animals were housed in plastic cages at room temperature and fed a conventional balanced diet ad libitum. Five to six mice per group were used for each time point tested.

### 4.3. Respiratory Infection with K. pneumoniae LABACER 01 and LABACER 27

Mice were lightly anesthetized and administered dropwise, through the nostrils, 100 μL of sterile PBS containing 10^7^ CFUs of *K. pneumoniae* LABACER 01 or LABACER 27. Mice in the control group were administered only 100 µL from PBS. The infective dose selected for this thesis work arose from experiments in which doses between 10^5^ and 10^9^ CFUs of *K. pneumoniae* LABACER 01 or LABACER 27 were evaluated (data not shown).

### 4.4. K. pneumoniae Counts in Lungs

*K. pneumoniae* LABACER 01 or LABACER 27 cell counts in the lungs were performed in mice sacrificed on day 2 post-infection, and their lungs were excised, weighed, and homogenized in 5 mL of sterile peptone water. Homogenates were appropriately diluted in BHI broth, plated in duplicate on blood agar, and incubated for 18 h at 37 °C. *K. pneumoniae* colonies were counted, and the results were expressed as log_10_ CFUs per gram of lung. Hemocultures were performed similarly, and the results were expressed as positive or negative.

### 4.5. Lung Damage

The albumin content was used as a measure to quantify the increased permeability of the alveolar–capillary barrier, and the activity of the intracellular enzyme lactate dehydrogenase, an indicator of cellular cytotoxicity, was determined in BAL samples [59]. The albumin content was determined colorimetrically based on albumin binding to bromocresol green using a Wiener-Lab albumin diagnostic kit. LDH activity, expressed as units per liter of BAL, was determined by measuring the formation of the reduced form of NAD^+^ using Wiener’s reagents and procedures (Wiener-Lab, Rosario, Argentina).

The lung wet:dry weight ratio was measured as previously described [37,38]. Briefly, mice were euthanized and exsanguinated, and their lungs removed, weighed, and dried in an oven at 55 °C for 7 days. After drying, the lungs were weighed again. The wet:dry weight ratio was then calculated as an index of intrapulmonary fluid accumulation, without correction for blood content.

### 4.6. Serum Cytokines and Bronchoalveolar Lavages

Blood samples were obtained by cardiac puncture and collected in heparinized tubes [36,60]. BAL samples were obtained according to the technique developed in the Laboratory Immunobiotechnology of CERELA-CONICET (San Miguel de Tucuman, Argentina) [38,61]. The trachea was exposed and intubated with a catheter, and then two sequential bronchoalveolar lavages were performed on each mouse by injecting sterile PBS. The recovered fluid was centrifuged for 10 min at 900× *g* and the fluid frozen at −70 °C for subsequent cytokine determinations. Tumor necrosis factor alpha (TNF-α), interferon gamma (IFN-γ), interleukin 10 (IL-10), IL-1β, IL-6, IL-17, KC, and monocyte chemoattractant protein 1 (MCP-1) concentrations were determined in serum and BAL samples using commercial ELISA kits. IFN-γ (Mouse IFN-gamma Quantikine ELISA Kit, sensitivity: 2 pg/mL), IL-10 (Mouse IL-10 Quantikine ELISA Kit, sensitivity: 5.2 pg/mL), IL-1β (Mouse IL-1β DuoSet ELISA, sensitivity: 1.5 pg/mL), IL-6 (Mouse IL-6 Quantikine ELISA Kit, sensitivity: 1.8 pg/mL), and IL-17 (Mouse IL-17 Quantikine ELISA Kit, sensitivity: 4.7 pg/mL) from R&D Systems (USA). TNF-α (Mouse TNF alpha ELISA Kit, sensitivity: 9.1 pg/mL), MCP-1 (Mouse MCP1 ELISA Kit, sensitivity: 0.487 pg/mL), and KC (Mouse KC ELISA Kit, sensitivity: 1.95 pg/mL) kits were obtained from Abcam (Cambridge, UK).

### 4.7. Comparative and Functional Genomics Studies

To perform comparative and functional genomics studies, the complete genomes of *K. pneumoniae* LABACER 01 and LABACER 27 were sequenced with the Illumina MiSeq platform (Illumina Inc., San Diego, CA, USA) at INDEAR-BIOCERES (Rosario, Argentina), using a 2 × 150 bp read length sequencing protocol as described previously [14]. The comparative analysis of the genomes of *K. pneumoniae* LABACER 01 and LABACER 27 was carried out using their genome sequences and the complete genome sequences and drafts of the same species available in databases (Table S3). For this purpose, the ROARY tool was used using default parameters. Identification and classification of orthologous genes, identification of strain-specific genes, and analysis of pan-genomes and core-genomes were performed. The presence of genes of interest (antimicrobial resistance, virulence factors) was assessed using functional annotation.

### 4.8. Phylogenomic Analysis and Virulence Factor Genes

Ribosomal Multilocus Sequence Typing (rMLST), a method developed for the classification of bacteria and archaea down to the strain level [62], was applied to the *Klebsiella* genomes; 32/51 genes encoding ribosomal protein subunits (rps) were recovered from the species *K. pneumoniae* and queried with the other *Klebsiella* genomes using the BLASTn algorithm [63]. The sequences were concatenated with Mafft software and used for phylogenomic reconstruction with RAxML software [64]. For the reconstruction of the tree, the GTR substitution model and 1000 bootstrap replications were used.

Virulence factors associated with *K. pneumoniae* infections were retrieved from the NCBI database and compared across genomes using the BLASTp algorithm. Only sequences with at least 70% similarity were considered homologous. Capsular typing was performed using Kaptive software [65].

### 4.9. Electron Microscopy

Culture broths of *K. pneumoniae* LABACER 01 and LABACER 27 were centrifuged for 5 min at 5000 rpm, and the pellet was collected and washed with PBS and then suspended in 0.1 mL of PBS. One ml of Karnovsky fixative, pH 7.2 (2.66% paraformaldehyde, 0.1 M sodium phosphate buffer, and 1.66% glutaraldehyde) was added to the collected pellet. The slightly homogenized samples in the fixative were kept in a refrigerator until they were treated for microscopy analysis. The samples were evaluated at the Integral Center for Electron Microscopy CIME at the Faculty of Agronomy and Zootechnics of the National University of Tucumán (UNT, Tucuman, Argentina). Scanning electron microscope (SEM) analysis was performed in a Zeiss model crossbeam 340 scanning electron microscope. Transmission electron microscope (TEM) analysis was performed in a Zeiss brand pound 120 transmission electron microscope.

### 4.10. Statistical Analysis

The experiments were performed in triplicate, and the results were expressed as the mean ± SD. Statistical analyses were performed using Prism 8.0 (GraphPad software, San Diego, CA, USA). Comparisons among multiple groups across multiple time points were performed using two-way ANOVA with Tukey’s multiple comparison post hoc test. Comparisons between two groups were performed using unpaired Student’s *t*-tests. Differences were considered significant at *p* < 0.05.

## 5. Conclusions

This study is the first to evaluate in vivo the virulence and the immune response to KPC-2-producing hypermucoviscous *K. pneumoniae* strains belonging to ST25, which have emerged as clinically significant pathogens in Northwest Argentina. In addition, bioinformatic genomic analysis provided data to reveal the genetic diversity in clinical isolates of hypermucoviscous carbapenem-resistant *K. pneumoniae* ST25. The genomic and in vivo studies carried out here indicate that *K. pneumoniae* LABACER 01 is more virulent and invasive than the LABACER 27 strain. The results also suggest that *K. pneumoniae* LABACER 27 would be more adapted to colonize the respiratory tract, inducing less inflammation to prevent its elimination by the immune system. Deeper studies on the genetic potential of multiresistant pathogenic microorganisms as well as their cellular and molecular interactions with the host are of fundamental importance to assess the association of certain virulence factors with the intensity of the inflammatory response, an association that could be extrapolated to the clinical evolution in patients. In this sense, this work explored the virulence profile based on genomic and in vivo studies of hypermucoviscous carbapenem-resistant *K. pneumoniae* ST25 strains, expanding the knowledge of the infection biology of the emerging ST25 clone in Argentina.

## Data Availability

Data are contained within the article.

## Supplementary Materials

The following supporting information can be downloaded at: https://www.mdpi.com/article/10.3390/ijms23137361/s1.
