# Supplementary material for: Genomic and Immunological Characterization of Hypermucoviscous Carbapenem-Resistant Klebsiella pneumoniae ST25 Isolates from Northwest Argentina
Source: Int J Mol Sci. 2022 Jul 1;23(13):7361. doi: 10.3390/ijms23137361 (PMC9266295; doi:10.3390/ijms23137361)
Supplement: Supplementary file 1 [file ijms-23-07361-s001.zip › ijms-1734443-supplementary.pdf]

Supplementary Table S1. Unique virulence factors genes detected in the genome of *K. pneumoniae* LABACER 01 compared to strains LABACER 27, NTUH-K2044, ATCC43816, and RFJ293.

**Gen**	**Protein**
ompC	Outer membrane porin C. One of the major outer membrane proteins
fimC1	Chaperone involved in fimbriae biosynthesis
sfaG1	S-type adhesins, which allow bacteria to adhere to eukaryotic cells
smfA	Fimbrial protein A involved in adhesion
hemN	S-adenosyl-1-methionine enzyme
gltB	B subunit that is part of the heterodimeric protein glutamate synthetase
papH	P pili protein subunits
papC	Outer membrane protein that allows assembly and secretion of pili
yfcS	Fimbrial chaperone protein encoded in the yfc operon
yfcR	Fimbrial chaperone protein encoded in the yfc operon
cbtA	Toxin of the CbtA-CbeA toxin-antitoxin system. CbtA directly interacts with the cell division proteins FtsZ and MreB and inhibits GTPase activity and GTP-dependent polymerization of FtsZ, as well as ATP-dependent polymerization of MreB
cbeA	cbeA encodes antitoxin protein that prevents the binding of the toxic protein cbtA (neutralizes the toxic effect of cbtA)
cyoA	The operon synthesizes subunit II that is part of the ubiquinol oxidase complex
cyoB	The operon synthesizes the I subunit that is part of the ubiquinol oxidase complex
tamA	It synthesizes a protein that, together with tamB, is inserted into the outer membrane
tamB	Synthesizes a protein that, together with tamA, is inserted into the outer membrane

Supplementary Table S2. Unique virulence factors genes detected in the genome of *K. pneumoniae* LABACER 27 compared to strains LABACER 01, NTUH-K2044, ATCC43816, and RFJ293.

Gen	Protein
vapB	The vapBC operon encodes the type II toxin/antitoxin system. vapB produces the DNA gyrase inhibitory toxin
traA	*It belongs to the tra operon, which synthesizes the relaxase enzyme, this is an endonuclease/topoisomerase that recognizes the oriT sequence*
bigR	Synthesizes repressor protein associated with persulfide-sensitive biofilm growth
bfpA	It is found within the bfp operon, and encodes for the protein that is responsible for localized adhesion in epithelial cells
higA	It is the antitoxin of HigB translation-dependent interferase mRNA toxin
rfaH	It controls the transcription of a specialized group of operons that direct the synthesis, assembly, and export of exopolysaccharides, F conjugation pilus, and hemolysin
copA	It synthesizes the carrier protein that removes the Cu cation from the bacteria and prevents its accumulation and its bactericidal effect
aroE	Protein involved in serum resistance
purH	Synthesizes the bifunctional enzyme PurH responsible for a step in the purine biosynthetic pathway
yadV2 *yadV3*	Yad pili chaperone proteins, involved in adhesion to the epithelium of the bladder
sfmC	It is part of the sfm operon that produces fimbrial surface structures. sfmC has chaperone activity
sfmD	It is part of the sfm operon that produces fimbrial surface structures. sfmD has chaperone activity.

Supplementary Table S3. *Klebsiella* strains with complete genomes sequenced used in this thesis work.

Strain	Capsular type	Accession number
Kp 1088	KL1	NJPF00000000
Kp 1	KL47	NJPM00000000
Kp NUHL24835	KL2	CP014004
Kp KP28872	KL149	JABFQQ000000000
Kp KP28873	KL149	JABFQR000000000
Kp 2	KL47	NJPL00000000
Kp 3	KL47	NJPK00000000
Kp 5	KL47	NJPJ00000000
Kp 7	KL47	NJPI00000000
Kp B1647	KL51	MCFO00000000
Kp B20038	KL30	MCFP00000000
Kp B20143	KL24	MCFQ00000000
Kp B5055	KL2	AQCG00000000
Kp BJ1-GA	KL2	CBTU000000000
Kp NTUH-K2044	KL1	AP006725.1
Kp RJA166	KL1	CP019047
Kp RJF293	KL2	CP014008
Kp SA1	KL2	CBTW000000000
Kp SH-1	KL47	PJPG00000000
Kp SWU01	KL47	CP018454
Kp T69	KL2	CBTV000000000
Kp TK421	KL20	CP045694
Kp Kp_whw	KL5	CAGKKT01
Kq A708	KL23	
Kq KqPF26	KL56	CP065838
Kv 8917	KL114	CP063403
Kv AT-22	KL134	CP001891

Kv FH-1	KL57	CP054254
Kp LABACER 01	KL118	JABXNY000000000
Kp LABACER 27	KL10	JABXNZ010000000
